# Vaping: Public Health, Social Media, and Toxicity

**DOI:** 10.2196/53245

**Published:** 2024-04-11

**Authors:** Yehao Sun, Prital Prabhu, Dongmei Li, Scott McIntosh, Irfan Rahman

**Affiliations:** 1 Department of Environmental Medicine, University of Rochester Medical Center Rochester, NY United States; 2 Department of Clinical & Translational Research University of Rochester Medical Center Rochester, NY United States; 3 Department of Public Health Sciences University of Rochester Medical Center Rochester, NY United States

**Keywords:** addiction, behavior, behaviors, device, devices, e-cigarette, e-cigarettes, effect, effects, e-liquid, flavors, nicotine, public health, social media, human health, toxicity, vape user, vape, vaper, vapers, vaping

## Abstract

This viewpoint aims to provide a comprehensive understanding of vaping from various perspectives that contribute to the invention, development, spread, and consequences of e-cigarette products and vaping. Our analysis showed that the specific characteristics of e-cigarette products as well as marketing strategies, especially social media marketing, fostered the spread of vaping and the subsequent effects on human health and toxicity. We analyzed the components of e-cigarette devices and e-liquids, including the latest variants whose impacts were often overlooked. The different forms of nicotine, including salts and freebase nicotine, tobacco-derived nicotine, tobacco-free nicotine, and cooling agents (WS3 and WS23), have brought more choices for vapers along with more ways for e-cigarette manufacturers to advertise false understandings and present a greater threat to vapers’ health. Our work emphasized the products of brands that have gained significant influence recently, which are contributing to severe public health issues. On the other hand, we also discussed in detail the toxicity of e-liquid components and proposed a toxicity mechanism. We also noticed that nicotine and other chemicals in e-liquids promote each other’s negative effects through the oxidative stress and inflammatory nuclear factor kappa-light-chain-enhancer of activated B cells (NF-κB) pathway, a mechanism leading to pulmonary symptoms and addiction. The impact of government regulations on the products themselves, including flavor bans or regulations, has been limited. Therefore, we proposed further interventions or harm reduction strategies from a public health perspective.

## Vaping: A Public Health Issue

Recently, e-cigarette use among the adolescent population has become a concerning public health challenge in the United States. The popularity of the relatively new invention of e-cigarettes reversed the nation’s effort to reduce tobacco use among youths in the past 30 years [1]. According to data collected in 2021 in the United States, 11% of young adults aged 18-24 years currently use e-cigarettes, and 24% of 12th-grade students reported having engaged in vaping activities in the past 30 days [1,2]. This public health issue is not limited to the United States, however, as the prevalence of past 30-day vaping among adolescents aged 12-16 years in 68 different countries worldwide was 9.2% (2021) [3]. This is especially concerning, as e-cigarette use among early-adolescent smokers is a predictor of any smoking and more frequent cigarette smoking in late adolescence [4]. Besides the impact on smoking behaviors, adolescents who vape can develop severe respiratory symptoms that may even require ventilation, and other symptoms, including gastrointestinal tract symptoms, coughing, and hypoxemia, can also arise throughout the body [5].

e-Cigarette use has become a worldwide public health issue that demands our attention. To gather information and present it to the public, this paper has taken literature from PubMed and Google Scholar into consideration and introduced the advantages and disadvantages of vaping based on current literature by including conflicting reports and potential biases on social media and public health.

## e-Cigarette Brands and Characteristics

Due to the public impact of e-cigarette use, it is important to understand the characteristics of popular e-cigarette products. According to the 2022 National Youth Tobacco Survey, Puff Bar (29.7%), Vuse (23.6%), JUUL (22%), SMOK (13.5%), NJOY (8.3%), Hyde (7.3%), and Blu (6.5%) were found to be the most popular brands among youth [6]. Another study by Ali et al [7] showed that during the 4-week period that ended December 25, 2022, the top 5 brands with the highest e-cigarette unit sales were Vuse, JUUL, Elf Bars (Funky Vape or EB Design), NJOY, and Breeze Smoke. Among the products from all the brands mentioned above, only products from SMOK were third-generation e-cigarettes (also known as mods, a type of highly customizable aerosol-generating devices that use e-liquids and have subohm tanks that allow for higher wattage), while all other products were fourth-generation e-cigarettes (also known as pod mods, a type of modifiable pod cartridges including vape bars that use nicotine salts and can come in different shapes). Most fourth-generation e-cigarettes introduced above contain salt-form tobacco-derived nicotine (TDN) with a concentration of ≤5% and a volume below 2 mL. The products from Hyde are unique in that some of them contain larger volumes (up to 10 mL) that support a higher number of puffs, and these products may contain tobacco-free nicotine (TFN). The characteristics of TDN and TFN are discussed in a subsequent section.

In 2023, however, several new brands became more popular, including Elf Bars (EB Design, Lost Mary, Funky Vape, BC5000, and BC 7000), all from iMiracle Associates (originated from Shenzhen, China), FLUM float bars, ESCO bars, and Tyson. All of the products within these brands are fourth-generation e-cigarettes with nicotine salts as the source of nicotine. All the products contain a larger volume (up to 13 mL) and thus more puffs (≥2500 puffs). Only some products from iMiracle contained TFN, while the other brands only used TDN.

Among all the popular brands, Elf Bars (Funky Vape or EB Design, now rechargeable with pods) from iMiracle requires the most attention due to worries of it becoming the next “JUUL” that produces massive public health issues (e-cigarettes by JUUL were extremely popular from 2017 to 2020 and caused massive public health issues) [8]. In the youth population in England, around 50% of past 30-day vapers reported the use of Elf Bars (EB Design) [9]. Researchers have also suggested that the increase in vaping frequency and the shifted interest toward disposable e-cigarettes among English vapers could have been driven by the popularity of Elf Bars (EB Design) [9]. Moreover, an analysis of data from the National Poison Data System showed that 60.8% of e-cigarette–associated cases in poison centers with reported brand information were related to Elf Bars (EB Design) [10]. Therefore, its popularity, its ability to change vapers’ vaping behaviors that were demonstrated by the shift in vapers’ interests, and its potential for causing severe health issues all make Elf Bars (EB Design) a dangerous brand from a public health perspective, and public health interventions should be applied to prevent another “JUUL” from emerging and causing severe issues.

In addition to the most popular brands on the market, another set of brands also requires our attention, despite not being as popular. Both freebase nicotine and nicotine salts are used in brands including EC Blend (uses TDN), Halo (TDN), Coastal Clouds (TDN), Bad Drips (TDN and TFN), Naked (TDN and TFN), Cloud Nurdz (TFN), and Primus (TDN); however, these compositions change regularly. e-Cigarettes originally used freebase nicotine, while more recent e-cigarettes started using nicotine salts, which have a lower pH and therefore are less irritating to the throat and allow for higher doses [11].

The difference between freebase nicotine and nicotine salts is not restricted to user experience, as different physiological and toxic properties were observed. Research in pharmacokinetics has shown that nicotine salts are absorbed more rapidly and can reach higher concentrations, while freebase nicotine is metabolized more slowly, causing it to remain at higher concentrations in male rats, which may be generalizable to humans [12]. As advertised, these characteristics allow vapers to experience an instant nicotine “hit” or a prolonged sensation, or they can even achieve both by using both types of products. However, the availability of both types of products in these brands allows vapers to encounter the different adverse effects of either type. Generally, nicotine salts produce changes in levels of more types of proinflammatory cytokines and have more complicated effects on human nasal epithelial cells, but freebase nicotine can also cause unique changes, including increased secretion of interleukin-7 [13]. The results above were yielded with the same nicotine concentration, while nicotine salt products often have double the nicotine concentration than freebase nicotine products [14]. Therefore, the toxicity of nicotine salt products should be more serious than that of freebase nicotine products, but the threat of the availability of freebase nicotine along with nicotine salt is also not neglectable.

## e-Liquids Composition, Toxicology, and Associated Pulmonary Symptoms or Responses

**Figure 1:**
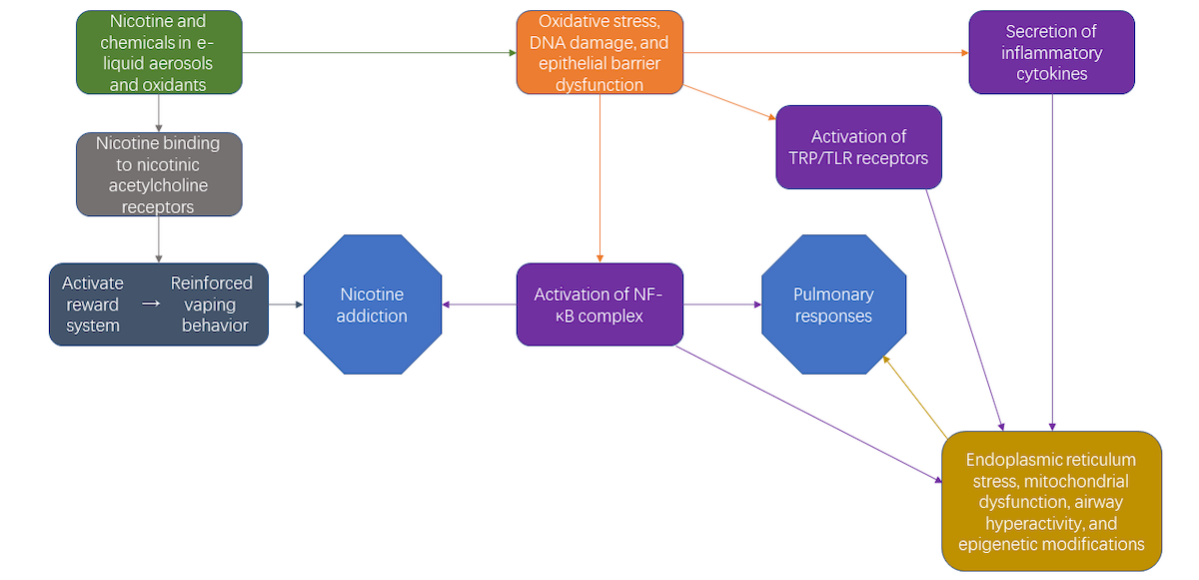
Biological pathways to pulmonary responses and nicotine addiction. NF-κB: nuclear factor kappa-light-chain-enhancer of activated B cells; TLR: toll-like receptors; TRP: transient receptor potential-like channels.

Besides the toxicities specific to nicotine salts, there are various toxic effects of almost all the components in the e-liquids [15-24]. The most abundant chemicals, propylene glycol, and vegetable glycerin, can negatively affect cell viability as they can cause decreases in cell growth to a similar degree to dimethyl sulfoxide [15]. Propylene glycol is known to cause respiratory arrest in rats after administration of 25 mg/kg/day for 3 days [16]. Chronic propylene glycol exposures are associated with reported symptoms of chronic wheezing, chest tightness, and weaker lung function, while acute exposures are associated with coughs and other upper airway symptoms as well as ocular irritations [17,18]. Heating vegetable glycerin in the presence of other acids may cause it to undergo pyrolysis reactions, releasing acrolein, which can cause nasal cavity irritations, lung lining damage, and even contribute to the onset of cardiovascular diseases [19-21]. Furthermore, the chemical that vapers are addicted to (ie, nicotine) can induce health issues by increasing reactive oxygen species, causing lipid peroxidation, and damaging human DNA [22] (Figure 1).

The most commonly used flavorants in e-liquids mentioned above also have pronounced toxicities [15,23,24]. Vanillin, the flavorant used in 35% of e-liquids, is positively correlated with the toxicity of the e-liquids (*R*^2^=0.62) [15]. Meanwhile, ethyl maltol leads to incidences of kidney lesions in rats and mild hemolytic anemia in dogs [23]. Ethyl butyrate, a type of ethyl ester flavor additive, can be broken down under high temperatures (may be achieved by atomizers) into carboxylic acids [24]. These carboxylic acids can then decompose into ketene, a chemical known to be a strong respiratory poison that can cause severe lung damage even at lower concentrations [24]. Generally, it is shown that the more chemicals there are in an e-liquid and vaporized (aerosols), the higher toxicity that e-liquid is likely to have [15].

Another group of deleterious chemicals that are present in e-liquids and aerosols are volatile organic chemicals (VOCs), including benzene, aldehydes, toluene, and ethylbenzene [25]. VOCs are significant risk factors for asthma among children [26]. In fact, the risk for asthma doubles for every 10 μg/m^3^ increase in the concentration of toluene, and it even triples for benzene [26]. Meanwhile, VOCs are also associated with reduced lung function in all age groups [27].

Besides the chemicals in the e-liquids themselves, vapers may inhale other toxic chemicals, including heavy metals (cadmium, lead, nickel, copper, arsenic, and chromium), while vaping and experience health risks, including increased oxidative stress, DNA damage, and decreased cellular viability in tissues [28,29]. It is observed that later puffs contain higher concentrations of toxic metals, and these higher concentrations can lead to a 30% increase in DNA damage after a 7-day exposure [28]. The commonly used flavorant, ethyl maltol, can also enhance the absorption and toxicity of copper in lung epithelial cells, as apoptosis, DNA damage, and oxidative stress occurred after coexposure to ethyl maltol and copper at low concentrations, which cannot initiate toxic effects individually [30]. Such toxicities can be applicable to most vapers, as higher cadmium, lead, silver, vanadium, nickel, and chromium concentrations were found in the urine or serum of e-cigarette users [31-33].

Due to the toxicity of these chemicals, although e-cigarettes are potentially less harmful than traditional cigarettes (less nitric oxide, oxidants, aldehydes, and no carbon monoxide inhaled), they are not as safe as expected by vapers [34]. Moreover, studies that demonstrated less harmful effects for e-cigarettes mostly focused on short-term effects, so whether the long-term health effects of e-cigarettes are also lower remains unclear [34]. More longitudinal studies should be conducted to confirm the long-term, chronic effects of e-cigarette use.

## Tobacco-Free and Tobacco-Derived Nicotine

In addition to the toxic effects brought by the presence of certain chemicals, the source of such chemicals may have an effect as well. The source of nicotine in e-liquids can be divided into 2 categories: TDN and TFN (nicotine that is not derived from tobacco plants). The type of nicotine originally used in e-cigarettes was TDN, but TFN started to be used in e-cigarettes since products containing TFN were not defined as tobacco products by the Federal Food, Drug, and Cosmetic Act and thus could evade regulations. e-Cigarette manufacturers have also advertised TFN as safer and with smoother flavors, causing vapers to believe that TFN has lower risks and to be willing to use and even pay more for TFN products [35]. Similarly, young adults have an overall positive perception of TFN products’ flavors, and those who are willing to try TFN products believe that TFN products are less addictive [36]. Fortunately, on April 14, 2022, new legislation took TFN into consideration and granted the Food and Drug Administration the authority to regulate TFN products, and TFN products are no longer a viable way to evade regulations [37].

Considering the chemical composition of TDN and TFN, the fundamental difference between them is chirality. The nicotine in TDN products is mostly S-nicotine (only 0.1%-1.2% is R-nicotine), while the nicotine in TFN products is a racemic mixture of R- and S-nicotine (50% R-nicotine and 50% S-nicotine) [38,39]. There is limited information on how the chirality of nicotine may impact its health effects, but it is stated that TFN is pharmaceutically pure and does not contain impurities found in TDN, including tobacco-specific nitrosamines, which may contribute to negative health effects [40,41]. Research has shown that TFN products of beverages or minty or iced (containing cooling agents, such as WS3 and WS23) flavors generate significantly less reactive oxygen species (ROS) than their TDN counterparts, which concurs with the statement earlier [42]. However, no significant difference was detected with fruity or tobacco flavors, indicating that flavorants also play a role in the process of generating ROS [42]. Therefore, more research should be done to confirm the difference posed by TFN itself, and cellular experiments are also crucial to understanding the inflammatory response of humans to the different forms of nicotine.

## Mechanism of Toxicity

Overall, the inhalation of e-liquid aerosols can lead to lung injuries, and Kaur et al [43] proposed mechanisms for this process. In this mechanism, the chemicals in the aerosol produce ROS in the lungs and induce oxidative stress, DNA damage, and epithelial barrier dysfunction. These cellular toxicities then trigger the transient receptor potential-like channels or toll-like receptors, activate the NF-κB complex, and stimulate the release of inflammatory cytokines, including interleukin-1 beta, interleukin-6, and tumor necrosis factor-alpha, which are all parts of inflammatory responses [43]. These inflammatory responses then manifest as symptoms by inducing endoplasmic reticulum stress, mitochondrial dysfunction, airway hyperactivity, epigenetic modifications, and other disease-developing mechanisms [43].

Besides the inflammatory aspects of the activation of the NF-κB complex, such activation can also contribute to the addiction to e-cigarettes [43-45]. The NF-κB complex is known to facilitate the positive reinforcing effect of drugs through reward sensitization in the nucleus accumbens, thus exacerbating the development and maintenance of nicotine addiction in vapers [44]. The major addiction development pathway, on the other hand, originates from the binding of nicotinic acetylcholine receptors with nicotine [45]. Signals are then sent to the reward system in the central nervous system, including the nucleus accumbens, to reinforce the behavior and eventually lead to addiction [45]. Figure 1 describes the known biological pathways of the toxicity of and addiction to e-cigarettes.

As a result of these pathways, the addiction potential of e-cigarettes is not lower than that of traditional cigarettes [46]. A study that used the Fagerström test for nicotine dependence showed that exclusive e-cigarette users (mean 3.5) have a nicotine dependence level more than 2 times higher than that of traditional cigarette smokers (mean 1.6) [46]. Dual users also demonstrated higher dependence when using e-cigarettes (mean 4.7) than when using traditional cigarettes (mean 3.2) [46]. The high addiction potential of e-cigarettes may make them unsuitable for being used as smoke cessation tools, especially in young adults who have a higher risk of addiction to e-cigarettes, as demonstrated in the study [46].

## Social Media and Vaping

In 2022, the number of social media users in the United States reached over 302 million. Around 90% of the US population used social media as of 2023. Social media platforms, such as Twitter, Reddit, Instagram, TikTok, YouTube, and Facebook, have become increasingly popular, especially among youth and young adults. Instagram and YouTube are the most broadly used social media platforms among youth in the United States, with 80% of youth using YouTube and 72% of youth using Instagram [47].

With the increasing popularity of social media platforms in the United States, e-cigarette companies and vape shops have aggressively marketed vaping products on social media [48-52]. Vaping marketing and promotion posts dominate vaping-related social media posts with more user engagement [47,50,52]. Social media accounts of e-cigarette companies or vape shops usually post well-designed pictures with vaping products, images linking vaping with luxury lifestyles, price promotions, discounts, and product giveaways [53-55]. Examples of extensive use of such strategies include the “Doit4juul” campaign on social media platforms, including Instagram and YouTube, initiated by the JUUL manufacturer, and the campaign largely contributed to the rapid growth of the brand [56]. They also sponsor influencers with many followers to help them market vaping products [57,58]. Around US $75 million (inflation-adjusted 2021) was spent on marketing vaping products by the vaping industry in the third quarter of 2019, including marketing and promotion on various social media platforms that reach most of the US population, including youth under 18 years [57,59,60]. The massive marketing and promotions of vaping products on social media resulted in the misperception of vaping as a harmless activity [58]. They also increased the risk of vaping initiation, especially among youth and young adults [61-63]. Research also found that the initiation of vaping is associated with subsequent cigarette smoking [64,65]. However, a recent epidemiological longitudinal survey study using Population Assessment of Tobacco and Health Wave 1-5 data (2013-2019) implicates that baseline vaping is not associated with subsequent cigarette smoking initiation in youth who have never smoked before after adjusting for behavior risk factors, such as alcohol, marijuana, and other tobacco product use [66]. Fortunately, the advertisements for combustible cigarettes are more restricted by the government, and combustible cigarette companies can only focus on marketing at the point of sale and product packaging [67,68].

Besides the massive marketing and promotion activities of vaping industries, the public also uses social media platforms to share their opinions and user experiences on vaping products [48,50,69]. Our longitudinal examination of the vaping flavors mentioned in 2.8 million Reddit posts from January 2013 to April 2019 showed that the top 2 flavors were fruit and sweet, consistent with previous survey results during a similar period [50]. A further examination of the association of vaping with health symptoms mentioned in the same Reddit posts showed a significant comentioning of fruit-flavored vaping products and cardiovascular symptoms [48]. A sentiment analysis of over 2.7 million vaping-related Twitter posts (tweets) from May 31 to August 22, 2019, found the fruit, mint, and sweet flavors were more positively perceived, and the beverage and tobacco flavors were more negatively perceived by the public [50]. A systematic review of the vaping-related social media studies from 2007 to 2017 found the major topics related to vaping on social media included the health effects of vaping, vaper testimony, benefits and risks associated with vaping, regulations of vaping products, and vaping as smoking cessation aids [69]. Being exposed to antivaping content on social media was associated with reduced vaping activities among young people [70].

Vaping-related social media posts could also be used to examine the impact of vaping product regulation policies on public attitudes toward vaping [71-73]. With the high prevalence of vaping in youth and young adults and the e-cigarette or vaping use-associated lung injury (EVALI) outbreak in 2019, many state governments and the FDA started to ban flavored vaping products.

Overall, social media plays an important role in vaping product marketing, promotion, and communication with the public about the potential harms of vaping. Regulations on social media marketing of vaping products can help reduce vaping initiation in youth and young adults. Meanwhile, social media platforms could also be used to provide education to the public about the potential harms of vaping and deliver vaping cessation interventions or harm reductions to reduce the uptake of vaping in youth and young adults. An example of such an application is the Real Cost e-Cigarette Prevention Campaign [74]. However, the low frequency of educational posts and the lack of appeal to youth social media users may impact the efficacy of the intervention, and more research should be done to address these issues and improve its efficacy [74].

## Health Effects of Vaping

Due to the recent and fast e-cigarette popularization with the contributions of social media, there is limited scientific evidence regarding its exact long-term effects. However, e-cigarette use has caused the onset of respiratory symptoms among consumers, labeled EVALI, the etiology of which involves the inhalation of vitamin E acetate (VEA) along with other chemicals emitted from e-cigarettes [75]. Patients with EVALI present to the hospital with sterile exogenous pneumonitis and the symptoms of cough, chest pain, dyspnea, nausea, gastrointestinal tract symptoms, fatigue, and fever [76]. Although the identification of VEA in bronchoalveolar lavage fluid is commonly associated with EVALI, sampling and identifying VEA from bronchoalveolar lavage fluid are not widely available, so the diagnosis of EVALI remains to be done through exclusion [77]. Nevertheless, as of 2020, almost 3000 cases of lung injury hospitalizations related to vaping have been reported in the United States [78]. Lung biopsies taken from 8 of these patients in various centers revealed acute lung injuries, including organizing pneumonia, diffuse alveolar damage, or interstitial inflammation [79]. In addition to respiratory damage, e-cigarette use can have effects elsewhere. In a recent study, researchers noticed an association between e-cigarette use and seizures in youth, perhaps due to the high levels of nicotine or flavoring chemicals inhaled when vaping—this finding also raises concerns about the impacts of e-cigarette use on brain development and other neurological complications in the youth population [80]. e-Cigarettes have been shown to produce an increase in blood pressure and aortic stiffness, lending themselves to further cardiovascular stress [81], and can also be potentially carcinogenic due to formaldehyde-releasing agent formation during the vaporization process [82] and high levels of nitrosamines present in e-cigarette flavorings [83].

If used as a cessation method for traditional smoking, e-cigarettes have the potential to limit traditional smoking. However, e-cigarette corporations’ decision to expand their consumer base past quitting smokers ushered in a new generation plagued by nicotine addiction. Since 1999, combustible nicotine intake has been steadily decreasing among high schoolers, down from an average of 5 days per month to only 1; however, after the popularization of e-cigarettes among school-aged children in 2015-2017 (coinciding with the release and rapid growth of the JUUL corporation), these numbers have begun to rise, once again approaching 5 days [84]. While nicotine addiction in youth may be lucrative for e-cigarette corporations, it perpetuates a cycle of toxicant inhalation, leading to the dangerous symptoms described above.

## Public Health Interventions Against Toxicity

The serious health effects and the wide spread of e-cigarettes led to the urgent need to address the public health issue. However, the sole motivation of the tobacco industry is to maximize profit, so it is unlikely it will ever be motivated to abandon profit and help address the public health issue of tobacco product use [85]. Historically and to this day, this profit has clearly been at the expense of public health. Such expenses include the use of aggressive tactics, including lobbying for industry-favorable laws and regulations [86], marketing directly and indirectly to youth [87], targeting many channels and subpopulations with evolving marketing strategies [88], and advanced public relations approaches to undermine and misrepresent evidence-based science by inflating scientific uncertainty to undercut public health initiatives and regulatory actions [85,89]. Many industries have subsequently adopted this game plan of disrupting normative science, which leads to an assertion of personal accountability for what are actually industrially generated health hazards [86,90].

Despite overall drops in rates of smoking (including mentholated products), menthol product use has increased, especially among young adults, female individuals, and Black users. Bans on menthol, a flavoring that has historically been disproportionately marketed to African American communities and associated with a lower likelihood of cessation among persons from these communities, were thought to have a high potential for increasing cessation in the communities [91]. Governments have taken steps to outlaw the retail sale of flavored tobacco products, including menthol cigarettes. The regulations primarily include the requirement of premarket tobacco product applications for e-cigarettes (as in traditional cigarettes), raising the age restriction of e-cigarette purchases to 21 years, broadening smoke-free policies and high taxation (originally only against traditional cigarettes) to e-cigarettes, and flavor bans [1]. These regulations target different aspects of e-cigarette use, but they all have imperfections in that the implementation of such regulations has a long processing time and not all states and jurisdictions would choose to implement such policies [1]. Therefore, e-cigarette sales are still largely underregulated.

On the other hand, the tobacco industry has once again aggressively fueled erroneous information about these policies, including the claim that such flavor bans target African American smokers’ freedom of choice, in service of their goals to protect profit [92]. The example demonstrated that the industries would exert all their effort and resources to oppose such prohibitions [92].

Telephone quitlines have demonstrated effectiveness for smoking cessation for more than 20 years [88]. They can produce both short-term and long-term intervention effects [88]. It has also been shown that promotions through television and radio have tripled call rates [88]. It is reasonable to infer that such promotions and best practices can be effective for vaping cessation, especially when promoted on social media. Since young adults and adolescents are the most vulnerable to nicotine addiction and the other negative effects of vaping, it is important to analyze the quitlines’ effectiveness in these understudied populations [89]. More research is needed to analyze the effectiveness of quitlines on adolescent and young adult vape users, including analysis of the effects of quitline-related treatment modalities such as websites, user chat rooms, and SMS text messaging. Additionally, it is needed to analyze the reach and efficacy of social media promotion of such eHealth intervention platforms.

## Summary and Conclusion

Overall, we have discussed the various aspects of vaping and its related toxicity. Social media marketing is an essential part of the companies’ strategies due to its high influence among youth. Their strategies are successful for their companies, as they gained substantial growth in their sales, while causing almost 3000 hospitalizations associated with vaping in the United States [78]. The most popular and most problem-causing brands discussed in the study should be monitored, especially Elf Bars (EB Design or Funky Vape or Funky Land), which is responsible for 60.8% of e-cigarette–associated cases in poison centers with reported brand information [10]. The toxicity mechanism of and addiction to e-liquids was also proposed in this study, including the chemicals’ contributions to nicotine addiction in vapers (Figure 1). More research should be done on the newer variants of e-liquid components to address the lack of understanding of these components and their potential to cause public health issues (ie, chronic effects on toxicities and health effects). In addition, a lack of knowledge about the long-term effects of vaping may undermine the determination of e-cigarette regulations, so longitudinal studies should be conducted to acquire the crucial information [34]. Investigations on the potential effectiveness of vaping cessation on the mitigation of both short- and long-term health effects would also be crucial in combating the vaping epidemic.

Considering the complexity of the toxicity that stemmed from the new e-liquid formula (bars and e-liquids with devices) and subsequent health issues, vaping cessation interventions or harm reduction should also address all these concerns. From a public health perspective, social media marketing of vaping products should be prohibited, and social media should instead be leveraged to provide education and cessation resources to the public. Government or federal regulations alone on flavored tobacco products may have a limited impact since corporations can bypass such regulations with their resources [87]. Viable intervention strategies should include telephone quitlines and related eHealth interventions, such as websites and SMS text messaging, which should be promoted to and tailored for adolescent and young adult populations.

## Abbreviations

EVALI: e-cigarette or vaping use-associated lung injury
NF-κB: nuclear factor kappa-light-chain-enhancer of activated B cells
ROS: reactive oxygen species
TDN: tobacco-derived nicotine
TFN: tobacco-free nicotine
VEA: vitamin E acetate
VOC: volatile organic chemical
